# Cropland encroachment detection *via* dual attention and multi-loss based building extraction in remote sensing images

**DOI:** 10.3389/fpls.2022.993961

**Published:** 2022-09-06

**Authors:** Junshu Wang, Mingrui Cai, Yifan Gu, Zhen Liu, Xiaoxin Li, Yuxing Han

**Affiliations:** ^1^College of Electronic Engineering, College of Artificial Intelligence, South China Agricultural University, Guangzhou, China; ^2^Shenzhen International Graduate School, Tsinghua University, Shenzhen, China

**Keywords:** UAV, cropland observation, building extraction, WHU building dataset, Massachusetts building dataset, multi-loss, dual attention, Sobel edge loss

## Abstract

The United Nations predicts that by 2050, the world’s total population will increase to 9.15 billion, but the per capita cropland will drop to 0.151°hm^2^. The acceleration of urbanization often comes at the expense of the encroachment of cropland, the unplanned expansion of urban area has adversely affected cultivation. Therefore, the automatic extraction of buildings, which are the main carriers of urban population activities, in remote sensing images has become a more meaningful cropland observation task. To solve the shortcomings of traditional building extraction methods such as insufficient utilization of image information, relying on manual characterization, etc. A U-Net based deep learning building extraction model is proposed and named AttsegGAN. This study proposes an adversarial loss based on the Generative Adversarial Network in terms of training strategy, and the additionally trained learnable discriminator is used as a distance measurer for the two probability distributions of ground truth *P_data_* and prediction *P*_*g*_. In addition, for the sharpness of the building edge, the Sobel edge loss based on the Sobel operator is weighted and jointly participated in the training. In WHU building dataset, this study applies the components and strategies step by step, and verifies their effectiveness. Furthermore, the addition of the attention module is also subjected to ablation experiments and the final framework is determined. Compared with the original, AttsegGAN improved by 0.0062, 0.0027, and 0.0055 on Acc, F1, and IoU respectively after adopting all improvements. In the comparative experiment. AttsegGAN is compared with state-of-the-arts including U-Net, DeeplabV3+, PSPNet, and DANet on both WHU and Massachusetts building dataset. In WHU dataset, AttsegGAN achieved 0.9875, 0.9435, and 0.8907 on Acc, F1, and IoU, surpassed U-Net by 0.0260, 0.1183, and 0.1883, respectively, demonstrated the effectiveness of the proposed components in a similar hourglass structure. In Massachusetts dataset, AttsegGAN also surpassed state-of-the-arts, achieved 0.9395, 0.8328, and 0.7130 on Acc, F1, and IoU, respectively, it improved IoU by 0.0412 over the second-ranked PSPNet, and it was 0.0025 and 0.0101 higher than the second place in Acc and F1.

## Introduction

**FIGURE 1:**
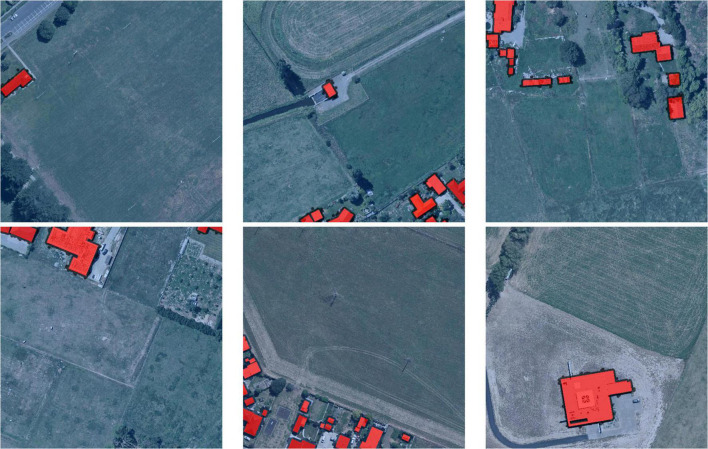
Encroachment by buildings on cropland.

Since 1990, the trend of population migration to cities has become more pronounced, which has resulted in cities becoming the main carriers for modern human economic and social activities (Buhaug and Urdal, 2013). Statistics show that the average global cropland area loss between 1992 and 2004 was about 30,000 km^2^yr^–1^, of which 34.3% was converted to settlements, and that cropland loss was particularly pronounced in Asia in the following decade (Tan and Li, 2019). Especially in China where, even though the illegal occupation of planting land has been written into the criminal law, the occupation of cropland is still common (Xing, 2016). Due to the rapid urbanization process, the occupation of cropland is often reflected in the expansion of building areas (as shown in Figure 1), which has become a common phenomenon (McKittrick, 2013). Therefore, the automatic detection of buildings is crucial to the protection of cropland. On the other hand, for automated agricultural intelligent devices such as robots and UAVs, accurate identification of buildings will also provide effective reference information for their path planning and obstacle avoidance tasks.

Buildings are one of the most widely distributed and most important types of man-made objects and could be extracted by satellite or UAV (Unmanned Aerial Vehicle) remote sensing images understanding (Alshehhi et al., 2017). Currently, with the development of remote sensing technology, such as SPOT 6 of France, ZY-3, Gaofen-1 and Gaofen-2 of China, and WorldView-3 of the United States can already use meters or submeters as its spatial resolution measurement unit, and it has reached or approached the quality of aerial photography (Chen W. et al., 2017; Ghimire et al., 2020). Compared with medium and low resolution, higher resolution remote sensing images have the following characteristics:

(1) The spectral features of the ground objects are more obvious, the spectral difference between the same type of ground objects becomes larger, and the spectral difference between different types of ground objects becomes smaller;

(2) Higher spatial resolution makes the data volume of a single image larger;

(3) A single pixel often corresponds to only one type of ground object;

(4) There is more detailed information of ground objects, such as shape, brightness, texture, etc;

(5) The background of ground objects is more complex and diverse.

These distinctive features also present higher requirements for building extraction. In order to meet these various requirements of new application areas, identifying buildings in high-resolution remote sensing images is the core challenge.

Traditional remote sensing image building extraction methods mainly include knowledge-based methods using geometric knowledge and context knowledge, along with object-based image analysis (OBIA)-based methods and machine-learning-based methods using image segmentation and target classification (Cheng and Han, 2016). In these traditional methods, the extraction task often requires experts to judge and design according to the spectrum, texture, shape, spatial relationship, and other information of the building, which relies heavily on abundant human imagination, ingenuity, and experience for the design of the features. Fortunately, Hinton and Salakhutdinov (2006) demonstrated the powerful feature representation capability of deep learning models in computer vision applications. They showed that the features will be automatically obtained from the existing data by the neural network through sampling, and the more abstract features beyond human imagination can be effectively obtained by increasing the depth of the network. The burden of feature design can be shifted to model design, which is relatively simple (Ubbens and Stavness, 2017).

However, in remote sensing images, due to the increasing complexity of buildings and their backgrounds caused by progressively higher resolution, the application of deep learning to building extraction still has problems (Jun et al., 2016), such as insufficient extraction of multi-scale targets, insufficient use of image information, model overfitting, and ambiguous edges in prediction, etc. Therefore, there are still challenges with regard to accurately segmenting and characterizing buildings. In this article, to solve these deficiencies, a deep-learning-based building extraction method is proposed. The contributions of this paper can be listed as follows:

(1) The dual-attention mechanism is used, which enhances the information utilization of remote sensing imagery within both feature maps and channels, and dedicates computing resources to more critical areas.

(2) In view of the multi-scale features of modern buildings, the ASPP (atrous spatial pyramid pooling) module is added to the model, which reduces the amount of computation and parameters while increasing the receptive field of the model, enhancing its ability to extract buildings with multiple sizes and shapes.

(3) In terms of model training, to make the prediction more artificial, a learnable discriminator and adversarial loss based on the idea of generative adversarial networks are proposed, and the authenticity of the prediction is used as an auxiliary reference to guide the learning process of the model by weighted adversarial loss.

(4) In terms of loss design, an edge loss based on the Sobel operator was proposed to solve the problem of the edges of buildings being susceptible to approximate background interference.

The following sections are arranged as follows: the relevant foundations involved in this study are presented in Section “Related works”; the components, WHU dataset, multi-losses design, evaluation indexes, etc., are detailed in Section “Materials and methods”; ablation experiments and comparative experiments are presented and discussed in Section “Results and discussion”; and in Section “Conclusion,” a summary of the full paper is given.

## Related works

### Image segmentation and semantic segmentation

The principle of building extraction is to use a building’s characteristics to achieve target recognition and accurately distinguish it from the background. Previous researchers tended to identify buildings in the order of image segmentation, and then artificial characterization (Khan, 2014). The traditional image segmentation method divides an image into several regions and realizes the feature similarity within the region and the feature difference between regions.

The main methods are: (1) the threshold-based segmentation method; (2) the edge-based segmentation method; (3) the region-based segmentation method; (4) the graph-based segmentation method; and (5) the energy-based segmentation method.

However, the above methods that utilize the low-level semantics do not fully utilize the high-level semantics of remote sensing images to qualitatively analyze the segmented regions. In practical application, especially when processing high-resolution images, the characteristics of targets will be relatively complex, and the differences between the same kinds of targets are relatively large; therefore, algorithms that only rely on low-level content information such as color, brightness, texture, etc., are insufficient to achieve a reasonable segmentation. Different from these traditional methods, the deep-learning-based semantic segmentation method can not only realize the image segmentation function, but can also achieve qualitative analysis and automatic classification for the area after clustering the pixels. In this process, abstract high-level semantic features will be fully utilized to achieve more accurate predictions.

The appearance of “semantic segmentation” as a noun can be traced back to the 1970s. Ohta et al. (1978) proposed the concept of semantic segmentation and emphasized assigning a label to each pixel in the image, thereby emphasizing the semantic meaning of the segmented region. Semantic segmentation belongs to the pixel-level scene understanding task in computer image processing, which enables a dense prediction of the input image and a label assignment for each pixel. Therefore, deep-learning-based semantic segmentation is not an isolated task, it involves image classification, target detection, target boundary division, etc., (Garcia-Garcia et al., 2017), which means it is a prediction task with high demands on image understanding.

The most meaningful models for the building extraction task in this study are fully convolutional networks (FCNs) and U-Net. In terms of task implementation, this study refers to the end-to-end idea of FCN. After the CNN (convolutional neural network) was proposed, researchers tried to apply its excellent learning performance to semantic segmentation tasks, for which the pioneering work is the FCN proposed by Long et al. (2015). FCN utilizes the powerful feature extraction capabilities of CNN to achieve end-to-end, pixel-to-pixel segmentation prediction and replaces traditional fully connected layers with convolutional layers. FCN also adapts classic network structures, such as AlexNet, VGG16, and GoogLeNet, to fully convolutional models and verifies their performance in semantic segmentation. In addition, FCN can accept input images of an arbitrary size with a fixed number and size of convolutional layers, and performs pixel-wise predictions on the input images through learnable deconvolution in terms of up-sampling.

After the performance of FCN is proved, more enlightening semantic segmentation models are proposed. Similar to U-Net in structure, PSPNet uses global pyramid pooling and deeply supervised loss as improvements, enhancing the ability of feature extraction. DenseASPP is proposed and used to solve the problem of insufficient feature resolution in the scale-axis. DANet proposes a dual-attention module that makes full use of image information and shows its performance in multi-class semantic segmentation. OCNet address the semantic segmentation task with a context aggregation scheme which focuses on enhancing the role of object information.

In this research, U-Net was referred to in the framework design. In U-Net, the contracting path performs the role of down-sampling, and the expansive path performs the role of up-sampling. It is worth noting that four connection channels were added, respectively concatenating the feature maps of four different resolutions in the down-sampling process with the corresponding layers in the up-sampling process. This operation avoids the loss of details in the down-sampling process, so that the shallow features extracted by the convolutional neural network can directly participate in the prediction.

In the process of down-sampling, the convolution calculation combined with the ReLU activation function plays a role in increasing the nonlinear relationship between pixels, and the image is shrunk by a 2 × 2 max pooling operation with a stride of two. After each contraction, the number of channels is doubled by a 3 × 3 convolution. After four contractions, U-Net starts to use a 2 × 2 convolution for expansion, and the number of channels will be reduced to half of the original through a 1 × 1 convolution and concatenated with the feature maps in contraction. Then, the number of channels of the output will be reduced by a 3 × 3 convolution with the ReLU function. It is worth noting that edge pixels will be lost after the convolution operation, so the corresponding feature map from the shrinking unit needs to be cropped before concatenation. Finally, U-Net will output the segmentation map according to the set size (Ronneberger et al., 2015).

### Generative adversarial networks

Before the proposal of GANs (generative adversarial networks), the deep learning model often included only a generative model or a discriminative model (Goodfellow et al., 2014). The former uses a large amount of neural network parameters and their ability to fit the dataset to generate new data that does not exist in the training set, while the latter directly fits the discriminant function. Different from the traditional model, GAN, as an implicit density generative model, includes both the generative model and the discriminative model in one framework. A generative model can be likened to a counterfeiter, while a discriminative model can be likened to a policeman. The former hopes that their forgery ability is as superb as possible, so that the fake data is as similar as possible to the real data, thus the police cannot make accurate judgments. The police, on the other hand, are expected to judge the authenticity of the data as accurately as possible, and the training process is more like a competition where the competitors are alternately leading. Assuming that *P*_*data*_(*x*) is the distribution probability of the real data and *P*_*g*_(*x*) is the distribution probability of the generated data, when the system is in Nash equilibrium, a “smartest” generator can be obtained to achieve more accurate fitting between *P*_*g*_(*x*) and *P*_*data*_(*x*) (Jabbar et al., 2021).

The advantage of GAN is that there are fewer constraints in the design, it does not need such a complex artificial qualification as that in the Markov chain or the variational boundary, but uses a learnable discriminator as an auxiliary training method to constrain the feature distribution of the generator output, which is more convenient. Moreover, the discriminator will act as a distance measurer between *P*_*g*_(*x*) and *P*_*data*_(*x*).

The generative adversarial networks can be expressed by the following object function:


(1) $$\min_{G}\max_{D}V\,\left(D,G\right)={𝔼}_{\text{x}∼{\text{P}}_{\text{data}}\,\left(\text{x}\right)}\,\left[\log\text{D}\,\left(\text{x}\right)\right]+{𝔼}_{\text{z}∼{\text{P}}_{\text{z}}\,\left(\text{z}\right)}\,\left[\log\left(1-\text{D}\,\left(\text{G}\,\left(\text{z}\right)\right)\right)\right]$$

where *D* represents the discriminator, *G* is the generator, *P*_*data*_(*x*) stands for the probability distribution of the real data, *P*_*z*_(*z*) denotes the probability distribution of random noise *z*, *D*(*x*) represents the discrimination result on real data *x*, and *D*(*G*(*z*)) signifies the discrimination result of *D* on sample *G*(*z*) generated by generator *G* through random noise *z*.

In terms of GAN training, according to the above principles, to obtain the optimal discriminator, it is necessary to let the output of *D*(*x*) be 1, and let the output of *D*(*G*(*z*)) be 0, then the optimal discriminator can be expressed as:


(2) $$\max_{D}V\,\left(D,G\right)={𝔼}_{\text{x}∼{\text{P}}_{\text{data}}\,\left(\text{x}\right)}\,\left[\log\text{D}\,\left(\text{x}\right)\right]+{𝔼}_{\text{z}∼{\text{P}}_{\text{z}}\,\left(\text{z}\right)}\,\left[\log\left(1-\text{D}\,\left(\text{G}\,\left(\text{z}\right)\right)\right)\right]$$

To obtain the optimal generator, it is necessary to let *G*(*z*) generate data as real as possible to disturb the judgment of the discriminator *D*. Since this process is independent of the first half of Formula (2), the optimal generator can be expressed as:


(3) $$\min_{G}V\,\left(D,G\right)={𝔼}_{\text{z}∼{\text{P}}_{\text{z}}\,\left(\text{z}\right)}\,\left[\log\left(1-\text{D}\,\left(\text{G}\,\left(\text{z}\right)\right)\right)\right]$$

To provide more accurate data for the subsequent city-related evaluation tasks, the building extraction has high requirements with regard to accuracy, and researchers hope the intensive prediction performance of the model can be as close as possible to human experts. Therefore, in this study, the training of the prediction model will be aided by weighted adversarial loss.

## Materials and methods

### Depthwise separable convolution and atrous spatial pyramid pooling

In recent years, the difference in shape and size between different buildings has become more pronounced; therefore, in remote sensing imaging, the identification and extraction of multi-scale objects has always been a challenge (Vakalopoulou et al., 2015). In a traditional convolution-based model, to increase the receptive field, reducing the amount of computation, pooling, or convolution with a stride greater than 1 will be used, but this will reduce the spatial resolution. In this study, ResNet-50 is used in the encoder; therefore, the depth of the model is relatively deep and the amount of parameters will be large (He et al., 2016). To ensure the resolution while expanding the receptive field, ASPP (atrous spatial pyramid pooling) and a depthwise separable convolution are used to obtain multi-scale information flexibly by setting the dilation rate without introducing additional parameters, so as to better obtain multi-size buildings.

Atrous spatial pyramid pooling was formally proposed in DeepLabv2. When deep convolutional neural networks are used in semantic segmentation tasks, the input remote sensing image usually needs to undergo a down-/up-sampling process in a convolutional encoder–decoder structure. Although convolutional neural networks have a receptive field mechanism that can be used to extract multi-scale target features, its scale will be limited by the size of the convolution kernel (Chen L. C. et al., 2017). An atrous convolution can be used to cheaply increase the receptive field of output units without increasing the kernel size, which is especially effective when multiple atrous convolutions are stacked one after the other (Dai et al., 2021). Assuming that the input feature map size is *R^in^*×*R^in^*, the output feature map size is *R^out^*×*R^out^*, and the convolution kernel size is *K*×*K*. In a traditional convolution, the receptive field range is equal to the size of the convolution kernel, which is *K*×*K*. In an atrous convolution, assuming that the dilated rate is *D*, its receptive field will be *K*′ = *K* + (*K*−1)(*d*−1).

### Loss function design

In this study, the overall loss is divided into three parts, namely BCE (binary cross-entropy) loss *L*_*bce*_ responsible for segmentation prediction, adversarial loss responsible for the auxiliary training of model prediction authenticity, and edge loss responsible for optimizing the accuracy of building edge prediction. The overall loss is defined as:


(4) $$L_{\mathrm{sum}}=L_{\mathrm{seg}}+L_{\mathrm{Edge}}+L_{\mathrm{adv}}$$

In the first item, the predicted segmentation map $\hat{y}$ and the label map *y* are compared at the pixel level. For a single pixel in a remote sensing image, the building extraction task belongs to the binary classification task; therefore, this study uses binary cross-entropy as the loss, which can be expressed as:


(5) $$L_{\mathrm{seg}}=L_{\mathrm{bce}}\,\left(\hat{y},y\right)=-\frac{1}{n}\,\sum_{i}^{n}z_{i}\,\log{\hat{z}}_{i}+\left(1-z_{i}\right)$$

where*z*_*i*_ and ${\hat{z}}_{i}$ denote the label value in *y* and the predicted value in $\hat{y}$ at the same location, respectively.

In the second item, considering that buildings often have straight boundaries with the background, in this study, a Sobel-operator-based loss was designed and added to highlight the edges. By implementing the Sobel operator in both horizontal and vertical directions, and then using it as a filter to perform convolution operations on the image to be processed, the horizontal and vertical edges on the image can be extracted. The Sobel template in the horizontal direction is:


(6) $$f_{h}=\left[\begin{array}{ccc} -1 & 0 & +1\\ -2 & 0 & +2\\ -1 & 0 & +1 \end{array}\right]$$

Meanwhile, in the vertical direction, it is:


(7) $$f_{v}=\left[\begin{array}{ccc} -1 & -2 & -1\\ 0 & 0 & 0\\ +1 & +2 & +1 \end{array}\right]$$

Specifically, two convolutional layers using the above templates are defined, and their weights are not involved in backpropagation. After the building extraction results are obtained in the forward propagation, the prediction results and the original labels are input into the two designed layers for calculation, and two dual-channel gradient maps of the edge are obtained, the values of which are between 0 and 1. Then, the mean square error (MSE) between the two gradient maps is calculated to obtain the edge loss:


(8) $$L_{\mathrm{Edge}}=L_{\mathrm{mse}}\,\left(f_{h}\,\left(y\right),f_{h}\,\left(\hat{y}\right)\right)+L_{\mathrm{mse}}\,\left(f_{v}\,\left(y\right),f_{v}\,\left(\hat{y}\right)\right)$$

In the third item, to ensure that the model prediction ability is closer to that of the experts, the idea of GAN is applied, and the extraction task is still carried out by the generator; meanwhile, an additional discriminator is trained synchronously to determine the authenticity of the pixel-level prediction results. Hence, the discriminator acts as a learnable constraint and participates in the overall training of the model by virtue of the adversarial loss, the training of which can be represented by the following function:


(9) $$L_{\mathrm{adv}}=L_{\mathrm{bce}}\,\left(D\,\left(x,y\right),1\right)+L_{\mathrm{bce}}\,\left(D\,\left(x,G\,\left(x\right)\right),0\right)$$

where $G\,\left(x\right)=\hat{y}$, *G* is the generator, and *D* represents the discriminator. In alternate iterative training of generative adversarial networks, the generator loss can be expressed as:


(10) $$L_{G}=L_{\mathrm{bce}}\,\left(\hat{y},y\right)+L_{\mathrm{Edge}}-L_{\mathrm{bce}}\,\left(D\,\left(x,G\,\left(x\right)\right),0\right)$$

Here, a maximized *L*_*bce*_(*D*(*x*,*G*(*x*)),0) can be equivalent to a minimized *L*_*bce*_(*D*(*x*,*G*(*x*)),1); furthermore, weights are added to the loss of each item, so it is easy to obtain:


(11) $$L_{G}=w_{1}\,L_{\mathrm{bce}}\,\left(\hat{y},y\right)+w_{2}\,L_{\mathrm{Edge}}+w_{3}\,L_{\mathrm{bce}}\,\left(D\,\left(x,G\,\left(x\right)\right),1\right)$$

### Dual-attention module

The aim of the attention mechanism is to obtain the difference in importance between feature maps and feature values. To realize reassignment, it causes the neural network to devote more computing resources to more important areas (Mi et al., 2020). In this building extraction task, the importance of different objects is distinct; therefore, introducing an attention module can provide more tractable and more relevant information for high-level perceptual reasoning and more complex visual processing tasks.

Generally, attention mechanisms can be divided into item-wise and location-wise, both of which can be subdivided into soft attention (differentiable), and hard attention (non-differentiable). Among them, the location-wise soft attention with feature map as an input can participate in gradient descent together with the neural network and update the weights through backpropagation (Niu et al., 2021), which is more suitable for the application scenario of deep learning, so it is also applied to this study.

In the process of building extraction, the spatial relationship between each pixel and its nearby pixels is significantly higher than the relationship with pixels far away from it; therefore, this study refers to DANet using a dual-attention module to fully capture the semantic dependencies in the spatial and channel dimensions (Fu et al., 2019).

**FIGURE 2:**
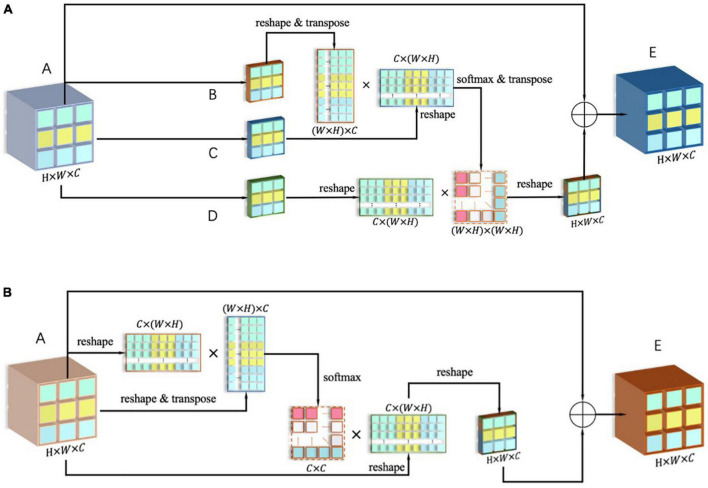
**(A)** Framework of position attention module; **(B)** framework of channel attention module.

In terms of implementation, the dual-attention module includes the position attention module [shown in Figure 2(A)] and the channel attention module [shown in Figure 2(B)], and calculates the attention matrices *S* and *X* for them, respectively.

First, the output *A* of the last layer after down-sampling is copied into four parts, in which *B*, *C*, and *D* are obtained after one convolution layer, and their size is {*B*,*C*,*D*} ∈ ℝ^C*H*W^. Subsequently, flattening is performed within the channel, and the new dimension is {*B*,*C*,*D*} ∈ ℝ^C*N^, where *N* = H*W. The reshaped matrix can be expressed as:


(12) $$B_{reshape}=C_{reshape}=D_{reshape}=\left[\begin{array}{ccc} \begin{array}{cc} M_{11}^{1} & M_{12}^{1}\\ M_{11}^{2} & M_{11}^{2} \end{array} & \cdots & \begin{array}{cc} M_{i\;j-1}^{1} & M_{i\;j}^{1}\\ M_{i\;j-1}^{2} & M_{i\;j}^{2} \end{array}\\ \vdots & \ddots & \vdots \\ \begin{array}{cc} M_{11}^{c-1} & M_{12}^{c-1}\\ M_{11}^{c} & M_{12}^{c} \end{array} & \cdots & \begin{array}{cc} M_{i\;j-1}^{c-1} & M_{i\;j}^{c-1}\\ M_{i\;j-1}^{c} & M_{i\;j}^{c} \end{array} \end{array}\right]\;$$

The matrix *B* is then transposed to get $B_{r\,e\,s\,h\,a\,p\,e}^{T}$, $B_{r\,e\,s\,h\,a\,p\,e}^{T}$ and *C* are multiplied by a matrix, and an attention matrix *pam* is formed with a size of N*N through the SoftMax, as shown in the following formula:


(13) $$S_{\mathrm{pam}}=\mathrm{softmax}\,\left(B_{\mathrm{reshape}}^{T}⊗C_{\mathrm{reshape}}\right)$$

It is then transposed, so that *pam^T^* and *D*_*reshape*_ are multiplied, and the *output* is then reorganized in the array dimension to make it the same as the input *A* ∈ ℝ^C*H*W^, which can be expressed as:


(14) $$\begin{array}{l} \text{​​​​​​​​​​​​​​​​​​​}output=D_{reshape}⊗S_{pam}^{T}=\\ \left[\begin{array}{ccc} M_{11}^{1}S_{11}+M_{12}^{1}S_{12}\ldots +M_{i\;j}^{1}S_{1n} & \cdots & M_{11}^{1}S_{n1}+M_{12}^{1}S_{n2}\ldots +M_{i\;j}^{1}S_{n\;n}\\ \vdots & \ddots & \vdots \\ M_{11}^{c}S_{11}+M_{12}^{c}S_{12}\ldots +M_{i\;j}^{c}S_{1n} & \cdots & M_{11}^{c}S_{n1}+M_{12}^{c}S_{n2}\ldots +M_{i\;j}^{c}S_{n\;n} \end{array}\right] \end{array}$$

In the *output* ∈ ℝ^C*H*W^ with an updated weight, each pixel in the original matrix is associated with the remaining pixels in the feature map (after being given new weight). Finally, *output* and *A* are added to get *E*, and it is used as the output of the spatial attention module.

In terms of the specific implementation of the channel attention module, the input *A* ∈ ℝ^C*H*W^ is first restructured into *A* ∈ ℝ^C*N^ (where *N* = H*W), and *A*_*reshape*_ is multiplied by its transposed $A_{r\,e\,s\,h\,a\,p\,e}^{T}$, then a SoftMax operation is performed on the result, and the channel attention map *X*_*cam*_ can be obtained, as shown in the formula below:


(15) $$X_{cam}=softmax\left(A_{reshape}⊗A_{reshape}^{T}\right)=\left[\begin{array}{ccc} \begin{array}{cc} S_{11} & S_{12}\\ S_{21} & S_{22} \end{array} & \cdots & \begin{array}{cc} S_{1c-1} & S_{1\;c}\\ S_{2\;c-1} & S_{2\;c} \end{array}\\ \vdots & \ddots & \vdots \\ \begin{array}{cc} S_{c-1\;1} & S_{c-1\;2}\\ S_{c\;1} & S_{c\;2} \end{array} & \cdots & \begin{array}{cc} S_{c-1\;c-1} & S_{c-1\;c}\\ S_{c\;c-1} & S_{c\;c} \end{array} \end{array}\right]\;$$

Next, the attention map *X* is transposed to obtain $X_{c\,a\,m}^{T}$, the transposed matrix is multiplied with *A*_*reshape*_ [as shown in Formula (16)], and the result is then reorganized into *output* ∈ ℝ^C*H*W^.


(16) $$\begin{array}{l} output=X_{cam}^{T}⊗A_{reshape}=\\ \left[\begin{array}{ccc} S_{11}M_{11}^{1}+S_{21}M_{11}^{2}\ldots +S_{c\;1}M_{11}^{c} & \cdots & S_{11}M_{i\;j}^{1}+S_{21}M_{i\;j}^{2}\ldots +S_{c\;1}M_{i\;j}^{c}\\ \vdots & \ddots & \vdots \\ S_{1\;c}M_{11}^{1}+S_{2\;c}M_{11}^{2}\ldots +S_{c\;c}M_{11}^{c} & \cdots & S_{1\;c}M_{i\;j}^{1}+S_{2\;c}M_{i\;j}^{2}\ldots +S_{c\;c}M_{i\;j}^{c} \end{array}\right] \end{array}$$

It is then added to input *A* to get output *E*. It can be seen from Formula (16) that the weights have been reassigned, and the new values are related to the values in the same position in all feature maps.

### Evaluation indexes

To evaluate the predictive ability of the model comprehensively and objectively, a confusion matrix is introduced in this study, which is used to summarize the predictive performance of classification models in machine learning.

Accuracy is used to find the portion of correctly classified values, and the formula is as follows:


(17) $$\mathrm{Accuracy}=\frac{\mathrm{TP}+\mathrm{TN}}{\mathrm{TP}+\mathrm{TN}+\mathrm{FP}+\mathrm{FN}}$$

where TP is True Positive, FP is False Positive, FN is False Negative, and TN is True Negative. Precision is used to calculate the model’s ability to classify positive values correctly, and the formula is as follows:


(18) $$\mathrm{Precision}=\frac{\mathrm{TP}}{\mathrm{TP}+\mathrm{FP}}$$

Recall is used to determine the model’s ability to predict positive value, and the formula is as follows:


(19) $$\mathrm{Recall}=\frac{\mathrm{TP}}{\mathrm{TP}+\mathrm{FN}}$$

The *F*1 score is a comprehensive analysis of whether the *TP* is large enough from two perspectives, predicted and actual. The *F*1 score is the harmonic mean of precision and recall. According to the formula of harmonic mean, it can be obtained by the following formula:


(20) $$F\,1={\left(\frac{{\mathrm{Precision}}^{-1}+{\mathrm{Recall}}^{-1}}{2}\right)}^{-1}$$

The formula for calculating IoU (intersection over union) is as follows:


(21) $$\mathrm{IoU}=\frac{\mathrm{TP}}{\mathrm{TP}+\mathrm{FP}+\mathrm{FN}}$$

### The model framework

**FIGURE 3:**
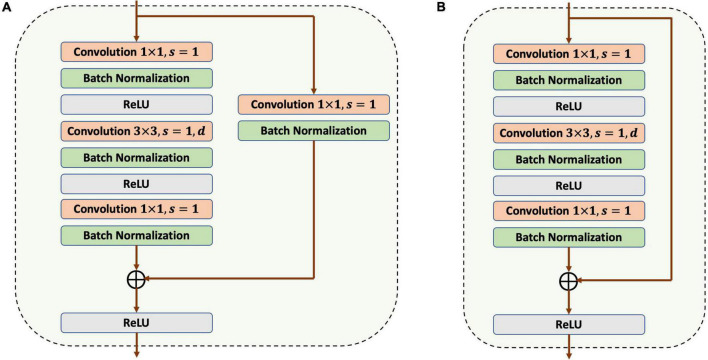
Framework of two types of bottlenecks. **(A)** Bottleneck 1. **(B)** Bottleneck 2.

In the framework design of the building extraction model, a convolutional encoder–decoder structure with skip connections was designed, as referred to U-Net and ResNet-50. In the down-sampling process, two slightly different bottlenecks are used, as shown in Figure 3, with the difference being that Bottleneck 1 contains a 1 × 1 convolutional and a BN in the shortcut connection.

**FIGURE 4:**
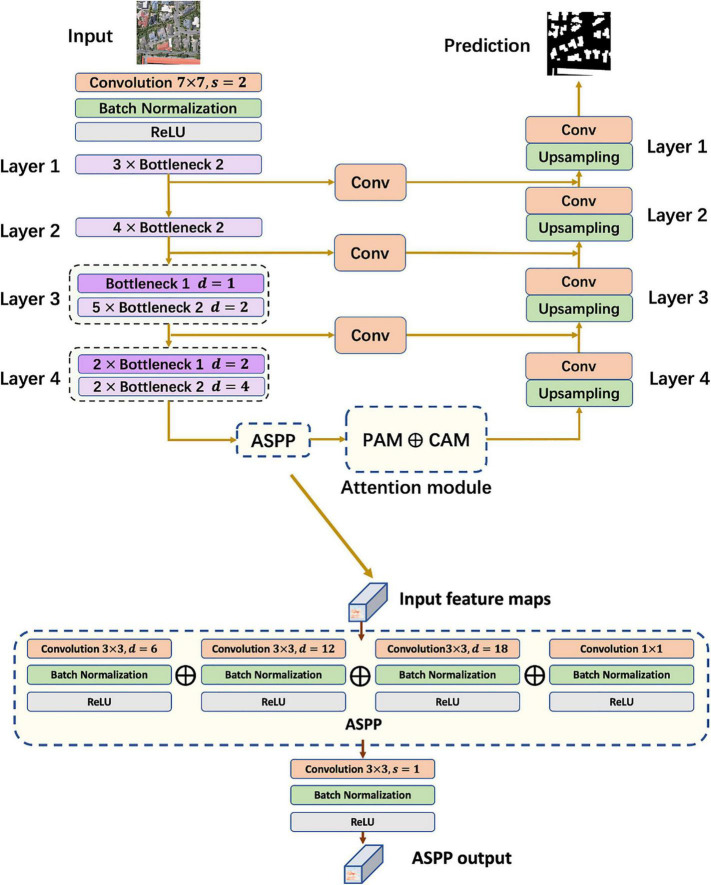
Framework of segmentation model and atrous spatial pyramid pooling.

In the convolutional encoder, the input image goes through four bottleneck blocks, and then enters ASPP. As shown in Figure 4, the ASPP module is divided into four parts, one of which is a normal 1 × 1 convolutional layer, and the remaining three set the dilation rate *D* to 6, 12, and 18, respectively. The output of the four parts is then concatenated and used as the final output after a 3×3 Conv+BN+ReLU operation. In the subsequent attention module, the input is reassigned according to the attention map and used as the input of the decoder. In the decoder, up-sampling is conducted using bilinear interpolation with convolutional layers, generating a prediction for building extraction.

**FIGURE 5:**
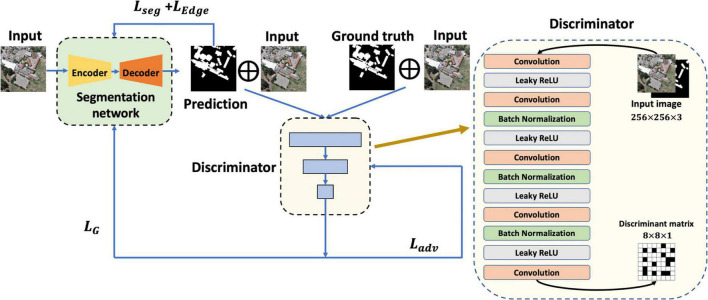
Markovian discriminator and gradient descent.

In terms of the discriminator structure, there are two different combinations of input: the first is the original image and the prediction, and the second is the combination of the original image and the ground truth. In this study, a Markovian discriminator (also known as PatchGAN) was designed with reference to Pix2Pix (Isola et al., 2017). The output of the discriminator is not a simple 1 or 0, but a discriminant matrix that gives a separate discrimination for each part of a grided image. To better judge the high-resolution remote sensing images with dense ground objects, the output size of the discriminator was set to 8 × 8 × 1, which is expected to output an all-zero matrix when judging the first combination, and an all-one matrix when judging the second combination. Figure 5 displays the Markovian discriminator and the process of gradient descent.

### Building datasets

**FIGURE 6:**
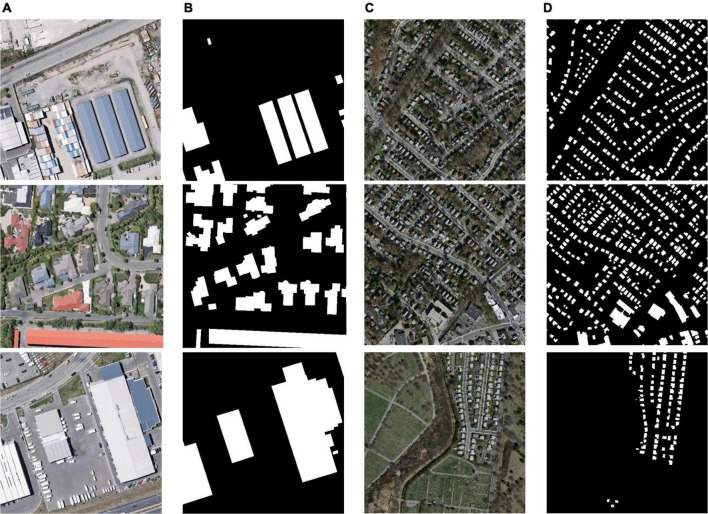
Images and labels in the WHU building dataset. **(A)** Original images in WHU dataset, **(B)** labels of WHU dataset, **(C)** original images in Massachusetts dataset, and **(D)** labels of Massachusetts dataset.

To verify the performance of the proposed model, two open-source building dataset was selected. WHU building dataset contains a total of 8,189 images, including 4,736 for training (containing 130,000 buildings), 1,036 for validation (containing 14,500 buildings), and 2,416 for testing (containing 42,000 buildings). This aerial dataset consists of more than 220,000 independent buildings extracted from aerial images with a 0.075°m spatial resolution covering 450°km^2^ in Christchurch, New Zealand. The area is divided into 8,189 blocks with a resolution of 512°×°512 each (shown in Figure 6). The WHU dataset contains a variety of scene types, such as countryside, residential, cultural, etc. The size, purpose, and color of the buildings are also diverse, which is suitable for the training of building extraction models (Ji et al., 2018).

The Massachusetts building dataset (shown in Figure 6) has a total of 137 remote sensing images, including 137 in the training set, four in the validation set, and 10 in the test set. The dataset covers buildings of different scales in cities and suburbs, the image size is 1,500°×°1,500 and the area is 2.25 square kilometers, the dataset covers about 340 square kilometers in total (Saito et al., 2016).

### Training details

The model was built in Pytorch v1.7.1, CUDA v11.1. The training equipment utilized was GeForce RTX 3090ti 24G, Adam was used as the optimizer, the learning rate was set to 0.001, and the momentum parameters were set to 0.9 and 0.999. The weights in the overall loss were set to *w*_1_:*w*_2_:*w*_3_ = 1:1:0.3. In the comparative experiments, each comparative model was trained for 200 epochs. It is worth highlighting that, to prevent the segmentation model from being excessively disturbed by the meaningless discrimination generated by the random initialized discriminator in the initial stage, AttsegGAN chose to freeze the discriminator first, and let the segmentation model train separately in the training set for 1,000 iterations with a batch size of 1. The segmentation model was then frozen, letting the discriminator train separately for 800 iterations of the combined input method described above. Then, the alternate iterative training strategy of the generative adversarial network was used to complete the subsequent training. The models used for comparison were trained according to the environmental parameters provided by the authors.

## Results and discussion

### Ablation experiments

To improve the prediction ability of the building extraction model, this study proposes four strategies based on an “hourglass” structure: U-Net (namely ASPP), attention mechanism, Sobel edge loss, and adversarial loss. To verify their effectiveness, this part of the experiment carried out ablation experiments in a step-by-step manner in WHU building dataset, and conducted objective evaluations through three evaluation indexes: Acc, F1, and IoU.

As shown in Table 1, after adopting the components and training strategies step by step, the prediction ability of the model was improved. Among them, the most significant improvement indicators were Acc and IoU; after adding all the improvement schemes, these two indicators were improved by 0.0062 and 0.0055, respectively, compared with the original version. The most obvious improvements to the model were adversarial loss and Sobel edge loss. After using the former, the IoU of the model was increased by 0.0038 and the F1 was increased by 0.0021, which means that the model could better predict positive values. The proposal of Sobel edge loss significantly improved the prediction ability, with an improvement of 0.0002 to 0.0021 in the evaluation indicators other than SP, and achieving the best results in the notable Acc, F1, and IoU, reaching 0.9875, 0.9435, and 0.8907, respectively. The improvement brought by the dual-attention mechanism was more significant in Acc, with an increase of 0.0059, indicating that the performance improved after the allocation of computing resources was adjusted through the attention map. Although the overall improvement brought by ASPP was relatively insignificant, it increased by 0.0012 to 0.8874 on IoU.

**TABLE 1:** Component and training strategy ablation experiments in WHU building dataset.

	Version 5 (proposed)	Version 4	Version 3	Version 2	Version 1
Sobel edge loss	✓				
Adversarial loss	✓	✓			
ASPP	✓	✓	✓		
Attention	✓	✓	✓	✓	
Acc	**0.9875**	0.9871	0.9867	0.9872	0.9813
F1	**0.9435**	0.9421	0.9400	0.9402	0.9408
IoU	**0.8907**	0.8905	0.8874	0.8862	0.8852

Bold values mean the best performing data. The underlined value means the second best performing data.

**FIGURE 7:**
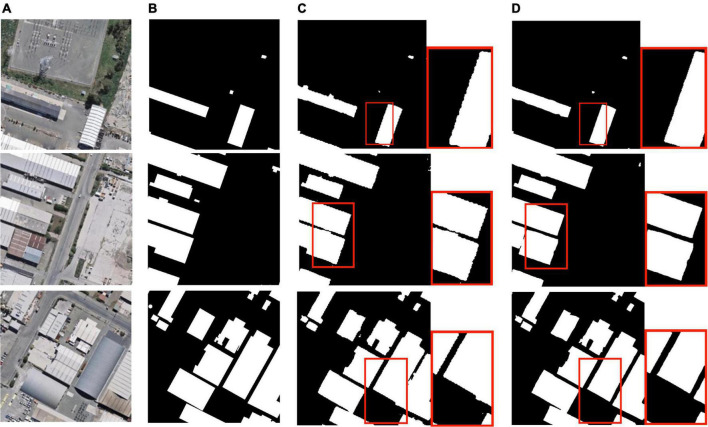
Building extraction results: **(A)** original remote sensing image; **(B)** ground truth; **(C)** prediction with atrous spatial pyramid pooling; **(D)** prediction with ASPP and Sobel edge loss.

Figure 7 shows the intuitive improvement brought by Sobel edge loss. It may not be able to improve the extraction of specific small-sized buildings, but it can make the lines of the extracted buildings clearer, making them closer to a straight line and to the ground truth. Although ASPP improved the extraction performance of the model in multi-size buildings while the evaluation indicators improved, it was also found that the edges of the buildings in the predicted segmentation map were obviously jagged due to the setting of the validation rate. Since semantic segmentation achieves pixel-level dense predictions, this phenomenon is not conducive to the prediction-accuracy-oriented task. However, Sobel edge loss used in conjunction with ASPP has been proven to effectively alleviate edge jaggedness.

### Attention mechanism ablation experiments

**FIGURE 8:**
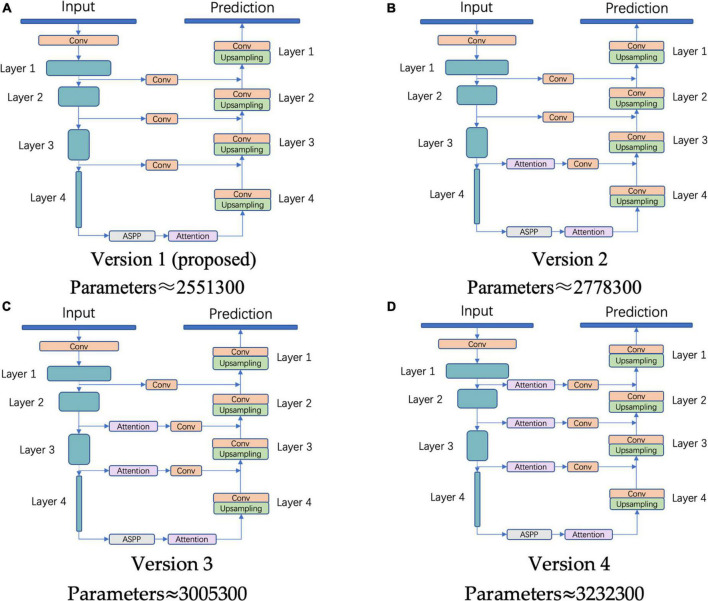
Attention mechanism ablation experiments. **(A)** Version 1 (proposed), **(B)** Version 2, **(C)** Version 3, and **(D)** Version 4.

According to our statistics, each time an attention module is added to the prediction model, approximately 227,000 parameters are added. Therefore, when the addition cannot effectively promote the capacity of prediction, it will increase the training cost and the risk of overfitting. In this section, the addition strategy of the attention mechanism is investigated and verified, and we propose several versions of the framework, as shown in Figure 8.

To explore the relationship between the attention module and the overfitting phenomenon, we performed the four versions on the WHU dataset and made statistics, as shown in Table 2.

**TABLE 2:** Statistics for the evaluation indicators of the three sets.

	Acc	F1	IoU
**Training set**			
Version 1	0.9869	0.9586	0.9107
Version 2	0.9873	0.9537	0.9119
Version 3	0.9870	0.9504	0.9039
Version 4	0.9878	0.9569	0.9153
**Validation set**			
Version 1	0.9883	0.8366	0.7889
Version 2	0.9879	0.8362	0.7870
Version 3	0.9889	0.8426	0.7898
Version 4	0.9883	0.8399	0.7903
**Test set**			
Version 1	**0.9875**	0.9435	**0.8907**
Version 2	0.9873	**0.9436**	0.8893
Version 3	0.9869	0.9421	0.8870
Version 4	0.9852	0.9397	0.8869

Bold values mean the best performing data.

From the performance on the test set, the predictive ability does not increase with the addition of the attention module, but decreases. Therefore, it is not advisable for this component to be added to the other connection channels; it works best when added only after the last down-sampling layer.

From Table 2, it can be found that Acc, F1, and IoU perform the best in the training set, indicating that after 200 epochs of training, the model can already learn enough and complete the prediction. Conversely, the indicators show a downward trend in the remaining two sets, and there is a large difference from the training set, indicating that these four versions have a certain degree of overfitting. This phenomenon is most obvious in Version 4, which has the largest number of parameters. Compared with the training set, the Acc, F1, and IoU of the model in the test set decreased by 0.0026, 0.0172, and 0.0284, respectively. Thus, although the attention mechanism has been proven to be an effective component, the improvement in predictive ability is not proportional to the number, and will lead to an aggravation of the overfitting, and thus performance degradation.

### Comparison with state-of-the-arts on WHU building dataset

In this section, we selected four classic semantic segmentation algorithms based on deep learning that have been proven in various open-source datasets: U-Net, DeepLabv3+, DANet, and PSPNet.

**FIGURE 9:**
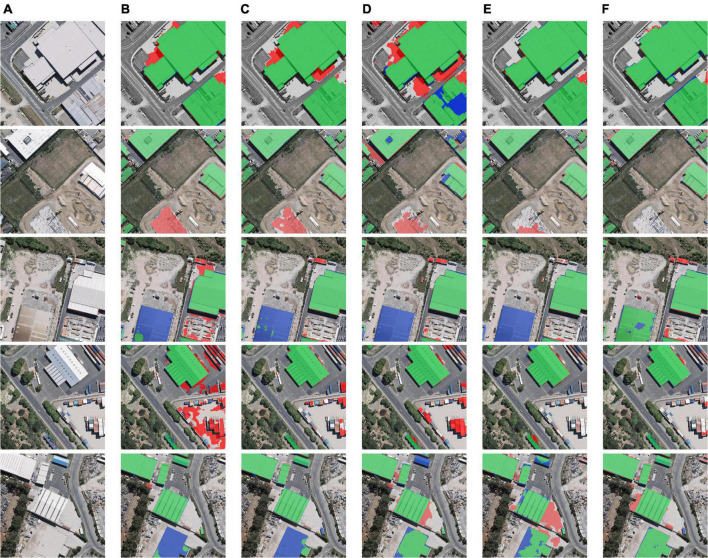
Building extraction results: **(A)** original remote sensing image; **(B)** prediction of U-Net; **(C)** prediction of DeepLabv3+; **(D)** prediction of PSPNet; **(E)** prediction of DANet; **(F)** prediction of AttsegGAN (ours). Green: true positive (tp) pixels; transparent: true negative (tn) pixels; red: false positive (fp) pixels; blue: false negative (fn) pixels.

As shown in Table 3, AttsegGAN is 0.1883 higher than U-Net in IoU, and 0.0260 higher in Acc, which indicates that the addition of effective components can improve the predictive ability of building extraction models in the case of similar deep learning frameworks. In comparison with DeepLabv3+ and DANet, AttsegGAN also has obvious improvement in indicators: 0.0099 and 0.0024, respectively, in ACC; and 0.0697 and 0.0169 in IoU, which proves that, even if the model uses components with similar principles, the rational framework and training strategy can also significantly improve the predictive ability of the building extraction model. The visual and intuitive results are shown in Figure 9, and the predicted segmentation results are objectively represented by rendering (images are randomly selected from the test set of the WHU building dataset).

**TABLE 3:** Statistics of comparative experiment results on WHU building dataset.

	Acc	F1	IoU
U-Net	0.9615	0.8252	0.7024
DeepLabv3+ (ResNet-101)	0.9776	0.9028	0.8228
PSPNet (ResNet-101)	0.9586	0.7734	0.6434
DANet (ResNet-101)	0.9851	0.9327	0.8738
AttsegGAN	**0.9875**	**0.9435**	**0.8907**

Bold values mean the best performing data. The underlined value means the second best performing data.

### Comparison with state-of-the-arts on massachusetts building dataset

To further demonstrate the predictive ability of the proposed AttsegGAN on pixel-level binary classification task, we trained and validated it on another remote sensing image based dataset, the Massachusetts building dataset. In this section, DANet, Deeplabv3+, PSPNet, and UNet were selected to compare with AttsegGAN.

**FIGURE 10:**
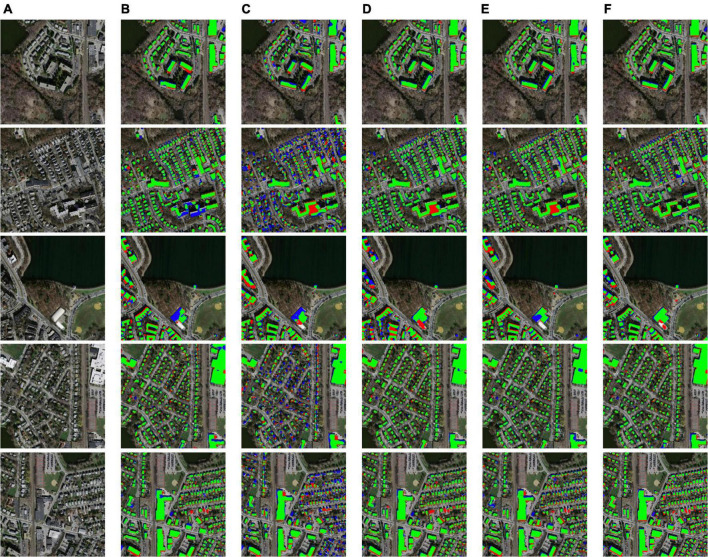
Building extraction results: **(A)** original remote sensing image; **(B)** prediction of U-Net; **(C)** prediction of DeepLabv3+; **(D)** prediction of PSPNet; **(E)** prediction of DANet; **(F)** prediction of AttsegGAN (ours). Green: true positive (tp) pixels; transparent: true negative (tn) pixels; red: false positive (fp) pixels; blue: false negative (fn) pixels.

From the statistics in Table 4, it can be seen that the performance of the models on the Massachusetts building dataset is lower than that on the WHU, but still reflects the difference between the prediction ability. In the evaluation indicators, AttsegGAN is higher than other algorithms in Acc, F1, and IoU. Among them, IoU is the most obvious, which is 0.0412 higher than the second-ranked PSPNet, this means that the predicted region fits the ground truth better. Meanwhile, AttsegGAN is also 0.0025 and 0.0101 higher than the second place in Acc and F1, respectively. It can be found that U-Net and AttsegGAN perform more prominently on Acc. As an earlier designed model, U-Net can outperform the newly proposed algorithm in binary classification task, indicating that the feature fusion brought by the skip connection mechanism can still effectively promote the prediction accuracy. The visual and intuitive results are shown in Figure 10, and the predicted segmentation results are objectively represented by rendering (images are randomly selected from the test set of the Massachusetts building dataset). In terms of running efficiency, when the input is a remote sensing image of size 512×512, the processing time of AttsegGAN is 0.09822s per image.

**TABLE 4:** Statistics of comparative experiment results on Massachusetts building dataset.

	Acc	F1	IoU
U-Net	0.9370	0.8125	0.6930
DeepLabv3+ (ResNet-101)	0.8921	0.6929	0.5301
PSPNet (ResNet-101)	0.9317	0.8227	0.6988
DANet (ResNet-101)	0.9236	0.7989	0.6652
AttsegGAN	**0.9395**	**0.8328**	**0.7130**

Bold values mean the best performing data. The underlined value means the second best performing data.

### Detecting buildings in cropland

**FIGURE 11:**
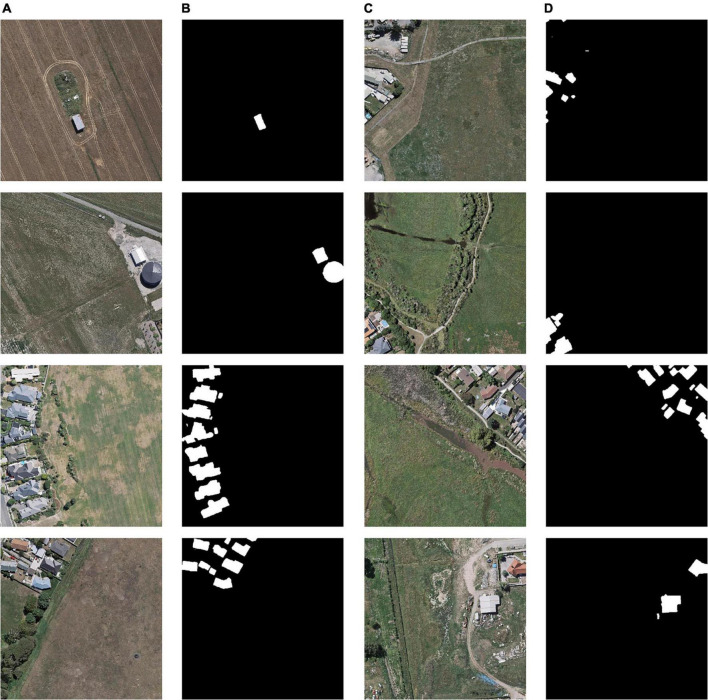
Building extraction results: **(A)** original remote sensing image; **(B)** prediction of AttsegGAN; **(C)** original remote sensing image; **(D)** prediction of AttsegGAN.

Recognition and background separation of buildings near planting land is a meaningful remote sensing image understanding task, which can provide significant reference information for planting land protection and path planning of unmanned equipment. In Figure 11, the processing performance of the proposed AttsegGAN on this task is visually displayed.

## Conclusion

Aiming to provide more accurate reference information for arable land monitoring tasks, AttsegGAN is proposed in this study. AttsegGAN is a deep-learning-based building extraction model that can automatically segment and characterize buildings from high-resolution remote sensing images. This study proposes four improvements based on the U-Net structure, namely ASPP and a dual-attention mechanism with regard to model components, and adversarial loss and Sobel edge loss with regard to training strategy, with experimentation carried out on the WHU building dataset. In the ablation experiments, the improvements were added one by one, and the effectiveness was proven on the test set using three evaluation indicators, Acc, F1, and IoU, with the results showing that the improvements brought by the two losses is more obvious. In the ablation experiments for the attention module, the results show that the model prediction ability is not positively related to the number of components, but leads to overfitting. In the comparison between the final version of AttsegGAN and state-of-the-arts, AttsegGAN performed the best in comparison with U-Net, DeepLabv3+, PSPNet, and DANet, achieving 0.9875, 0.9435, and 0.8907 for Acc, F1, and IoU in the WHU test set, respectively. Meanwhile, AttsegGAN also achieved the best results on the Massachusetts test set, achieving 0.9395, 0.8328, and 0.7130 for Acc, F1, and IoU. The results show that the proposed model could accurately complete building extraction and provide more reliable reference information for remote sensing observation tasks related to cropland.

## Data availability statement

Publicly available datasets were analyzed in this study. This data can be found here: http://gpcv.whu.edu.cn/data/building_dataset.html; https://www.cs.toronto.edu/~vmnih/data/.

## Author contributions

JW designed the experiments. JW and YH wrote the manuscript. MC and YH collected the data and analyzed the results. YG, ZL, and XL helped carry out the experiments. YH revised the manuscript. All authors contributed to the article and approved the submitted version.

## References

[B1] Alshehhi R. Marpu P. R. Woon W. L. Dalla Mura M. (2017). Simultaneous extraction of roads and buildings in remote sensing imagery with convolutional neural networks. *ISPRS J. Photogramm. Remote Sens.* 130 139–149. 10.1016/j.isprsjprs.2017.05.002

[B2] Buhaug H. Urdal H. (2013). An urbanization bomb? Population growth and social disorder in cities. *Glob. Environ. Change* 23 1–10. 10.1016/j.gloenvcha.2012.10.016

[B3] Chen L.-C. Papandreou G. Kokkinos I. Murphy K. Yuille A. L. (2017). Deeplab: Semantic image segmentation with deep convolutional nets, atrous convolution, and fully connected crfs. *IEEE Trans. Pattern Anal. Mach. Intell.* 40 834–848. 10.1109/TPAMI.2017.2699184 28463186

[B4] Chen W. Li X. He H. Wang L. (2017). A review of fine-scale land use and land cover classification in open-pit mining areas by remote sensing techniques. *Remote Sens.* 10:15. 10.3390/rs10010015

[B5] Cheng G. Han J. (2016). A survey on object detection in optical remote sensing images. *ISPRS J. Photogramm. Remote Sens.* 117 11–28. 10.1016/j.isprsjprs.2016.03.014

[B6] Dai F. Wang F. Yang D. Lin S. Chen X. Lan Y. (2021). Detection Method of Citrus Psyllids With Field High-Definition Camera Based on Improved Cascade Region-Based Convolution Neural Networks. *Front. Plant Sci.* 12:816272. 10.3389/fpls.2021.816272 35140732 PMC8819152

[B7] Fu J. Liu J. Tian H. Li Y. Bao Y. Fang Z. (2019). “Dual attention network for scene segmentation,” in *Proceedings Of The IEEE/Cvf Conference On Computer Vision And Pattern Recognition*, (Piscataway: IEEE), 3146–3154. 10.1109/TNNLS.2020.3006524

[B8] Garcia-Garcia A. Orts-Escolano S. Oprea S. Villena-Martinez V. Garcia-Rodriguez J. (2017). A review on deep learning techniques applied to semantic segmentation. *arXiv* [Preprint]. 10.48550/arXiv.1704.06857 35895330

[B9] Ghimire P. Lei D. Juan N. (2020). Effect of image fusion on vegetation index quality—a comparative study from Gaofen-1, Gaofen-2, Gaofen-4, Landsat-8 OLI and MODIS Imagery. *Remote Sens.* 12:1550. 10.3390/rs12101550

[B10] Goodfellow I. Pouget-Abadie J. Mirza M. Xu B. Warde-Farley D. Ozair S. (2014). Generative adversarial nets. *Adv. Neural Inf. Process. Syst.* 3:27.

[B11] He K. Zhang X. Ren S. Sun J. (2016). “Deep residual learning for image recognition,” in *Proceedings Of The IEEE Conference On Computer Vision And Pattern Recognition*, 10.1109/CVPR.2016.90 (Piscataway: IEEE), 770–778.

[B12] Hinton G. E. Salakhutdinov R. R. (2006). Reducing the dimensionality of data with neural networks. *Science* 313 504–507. 10.1126/science.1127647 16873662

[B13] Isola P. Zhu J.-Y. Zhou T. Efros A. A. (2017). “Image-to-image translation with conditional adversarial networks,” in *Proceedings Of The IEEE Conference On Computer Vision And Pattern Recognition*, 10.1109/CVPR.2017.632 (Honolulu: IEEE), 1125–1134.

[B14] Jabbar A. Li X. Omar B. (2021). A survey on generative adversarial networks: Variants, applications, and training. *ACM Comput. Surv.* 54 1–49. 10.1145/3463475

[B15] Ji S. Wei S. Lu M. (2018). Fully convolutional networks for multisource building extraction from an open aerial and satellite imagery data set. *IEEE Trans. Geosci. Remote Sens.* 57 574–586. 10.1109/TGRS.2018.2858817

[B16] Jun W. Qiming Q. Xin Y. Jianhua W. Xuebin Q. Xiucheng Y. (2016). A Survey of Building Extraction Methods from Optical High Resolution Remote Sensing Imagery. *Remote Sens. Technol. App.* 31 653–662.

[B17] Khan M. W. (2014). A survey: Image segmentation techniques. *Int. J. Futur. Comput. Commun.* 3 89–93. 10.7763/IJFCC.2014.V3.274

[B18] Long J. Shelhamer E. Darrell T. (2015). ““Fully convolutional networks for semantic segmentation”,” in *Proceedings Of The IEEE Conference On Computer Vision And Pattern Recognition*, 10.1109/CVPR.2015.7298965 (Piscataway: IEEE), 3431–3440. 27244717

[B19] McKittrick K. (2013). Plantation futures. *Small Axe* 17 1–15. 10.1215/07990537-2378892

[B20] Mi Z. Zhang X. Su J. Han D. Su B. (2020). Wheat stripe rust grading by deep learning with attention mechanism and images from mobile devices. *Front. Plant Sci.* 11:558126. 10.3389/fpls.2020.558126 33013976 PMC7509068

[B21] Niu Z. Zhong G. Yu H. (2021). A review on the attention mechanism of deep learning. *Neurocomputing* 452 48–62. 10.1016/j.neucom.2021.03.091

[B22] Ohta Y.-I. Kanade T. Sakai T. (1978). “An analysis system for scenes containing objects with substructures,” in *Proceedings of the Fourth International Joint Conference on Pattern Recognitions*, (Piscataway: Institute of Electrical and Electronics Engineers Incorporated), 752–754.

[B23] Ronneberger O. Fischer P. Brox T. (2015). “U-net: Convolutional networks for biomedical image segmentation,” in *International Conference On Medical Image Computing And Computer-Assisted Intervention*, (Berlin: Springer), 234–241. 10.1007/978-3-319-24574-4_28

[B24] Saito S. Yamashita T. Aoki Y. (2016). Multiple object extraction from aerial imagery with convolutional neural networks. *Electron. Imaging* 2016 1–9. 10.2352/ISSN.2470-1173.2016.10.ROBVIS-392

[B25] Tan M. Li Y. (2019). Spatial and temporal variation of cropland at the global level from 1992 to 2015. *J. Resour. Ecol.* 10 235–245. 10.5814/j.issn.1674-764x.2019.03.001

[B26] Ubbens J. R. Stavness I. (2017). Deep plant phenomics: A deep learning platform for complex plant phenotyping tasks. *Front. Plant Sci.* 8:1190. 10.3389/fpls.2017.01190 28736569 PMC5500639

[B27] Vakalopoulou M. Karantzalos K. Komodakis N. Paragios N. (2015). “Building detection in very high resolution multispectral data with deep learning features,” in *2015 IEEE International Geoscience And Remote Sensing Symposium (IGARSS)*, 10.1109/IGARSS.2015.7326158 (Piscataway: IEEE), 1873–1876.

[B28] Xing L. (2016). The Judicialize the Eco-Civilization Policy in China: A Perspective of Grasslands Protection. *Kan. J. L. Pub. Pol.* 26:396.

